# HIV protease inhibitor-induced cardiac dysfunction and fibrosis is mediated by platelet-derived TGF-β1 and can be suppressed by exogenous carbon monoxide

**DOI:** 10.1371/journal.pone.0187185

**Published:** 2017-10-31

**Authors:** Jeffrey Laurence, Sonia Elhadad, Tyler Robison, Hunter Terry, Rohan Varshney, Sean Woolington, Shahrouz Ghafoory, Mary E. Choi, Jasimuddin Ahamed

**Affiliations:** 1 Division of Hematology and Medical Oncology, Weill Cornell Medical College (WCMC), New York, New York, United States of America; 2 Cardiovascular Biology Research Program, Oklahoma Medical Research Foundation (OMRF), Department of Biochemistry and Molecular Biology, University of Oklahoma Health Science Center, Oklahoma City, Oklahoma, United States of America; 3 Division of Nephrology and Hypertension, Weill Cornell Medical College (WCMC), New York, New York, United States of America; University of Louisville, UNITED STATES

## Abstract

Human immunodeficiency virus (HIV) infection is an independent risk factor for cardiovascular disease. This risk is magnified by certain antiretrovirals, particularly the protease inhibitor ritonavir, but the pathophysiology of this connection is unknown. We postulated that a major mechanism for antiretroviral-associated cardiac disease is pathologic fibrosis linked to platelet activation with release and activation of transforming growth factor (TGF)-β1, and that these changes could be modeled in a murine system. We also sought to intervene utilizing inhaled carbon monoxide (CO) as proof-of-concept for therapeutics capable of regulating TGF-β1 signaling and collagen autophagy. We demonstrate decreased cardiac function indices, including cardiac output, ejection fraction and stroke volume, and prominent cardiac fibrosis, in mice exposed to pharmacological doses of ritonavir. Cardiac output and fibrosis correlated with plasma TGF-β1 levels. Mice with targeted deletion of TGF-β1 in megakaryocytes/platelets (PF4CreTgfb1^flox/flox^) were partially protected from ritonavir-induced cardiac dysfunction and fibrosis. Inhalation of low dose CO (250ppm), used as a surrogate for upregulation of inducible heme oxygenase/endogenous CO pathways, suppressed ritonavir-induced cardiac fibrosis. This occurred in association with modulation of canonical (Smad2) and non-canonical (p38) TGF-β1 signaling pathways. In addition, CO treatment suppressed the M1 pro-inflammatory subset of macrophages and increased M2c regulatory cells in the hearts of RTV-exposed animals. The effects of CO were dependent upon autophagy as CO did not mitigate ritonavir-induced fibrosis in autophagy-deficient LC3^-/-^ mice. These results suggest that platelet-derived TGF-β1 contributes to ritonavir-associated cardiac dysfunction and fibrosis, extending the relevance of our findings to other antiretrovirals that also activate platelets. The anti-fibrotic effects of CO are linked to alterations in TGF-β1 signaling and autophagy, suggesting a proof-of-concept for novel interventions in HIV/antiretroviral therapy-mediated cardiovascular disease.

## Introduction

HIV infection is an independent risk factor for cardiovascular disease (CVD) [1]. Its incidence is elevated in HIV-infected individuals receiving certain antiretroviral therapies (ART), with a relative risk for advancing carotid artery intima-medial thickness, a subclinical marker for atherosclerosis, of 13.6 for those exposed to HIV protease inhibitors (PI) [2] The PI ritonavir (RTV) has a strong correlation with CVD. Duration of RTV-boosted PI treatment was the only significant association for CVD among HIV-infected adolescents [3]. In addition, biomarkers for CVD risk that are elevated following RTV initiation often fail to normalize following its discontinuation [4,5]. Pathophysiology-based interventions are clearly required.

There are multiple pathways by which HIV/ART could promote CVD; we investigated cardiac fibrosis and its link to antiretroviral medications for three reasons:

1. Arterial inflammation directly correlates with biomarkers of inflammation and monocyte activation, but not with markers of HIV activity, in HIV-infected individuals on ART [6].
2. Computed tomographic angiography reveals prominent aortic arterial fibrosis in HIV-infected individuals vs. HIV negative controls, changes present in the ART-naïve and not suppressed by ART [7].
3. Plasma levels of transforming growth factor (TGF)-β1, a key regulator of fibrosis, are increased 2-fold in HIV+, ART-naïve asymptomatic individuals, with a further rise following advancing disease, and this is not suppressed by ART [8].

In terms of animal models, simian immunodeficiency virus-infected macaques show cardiac dysfunction similar to that of HIV infection, in association with interstitial and vascular fibrosis and macrophage infiltration of cardiac tissue [9,10]. With respect to ART, very high doses of RTV induced left ventricular (LV) fibrosis and systolic dysfunction in LBNF1 rats, but the concentrations employed, 75mg/kg daily, are over 7-fold higher than used clinically [11]. Pharmacologically appropriate concentrations of the RTV-boosted PI lopinavir (steady state levels of 7.1 ±2.9 μg/ml by constant infusion) did cause cardiac contractile dysfunction in Wistar rats after 8 weeks of treatment [12], which was attributed to lopinavir/RTV-mediated increases in serum LDL-cholesterol levels and perturbations in calcium handling, in the absence of detectable fibrosis [12]. In contrast, RTV did cause cardiac fibrosis at pharmacologically appropriate doses (5mg/kg) in ApoE^-/-^ mice, and this was independent of alterations in lipid metabolism [13]. However, by the methodology used in those experiments, only a trend toward increased TGF-β1 in plasma and cardiac tissue could be documented [13].

We specifically focused on platelet-derived TGF-β1 in a study of ART-linked cardiac dysfunction and fibrosis because: platelets contain 40–100 times the concentration of TGF-β1 as other cells; it is rapidly released upon platelet activation; it is a major component of circulating TGF-β1; and it contributes to cardiac fibrosis in a mouse model of heart failure [14,15]. In addition, platelet activation is characteristic of untreated HIV infection [16], and it persists or increases in the presence of several ART regimens [17,18]. We recently reported that pharmacologic concentrations of RTV activate platelets, inducing a 2-fold increase in TGF-β1 secretion from human platelet-rich plasma [18].

In terms of mechanisms relating RTV to TGF-β1 processing and fibrosis, it is important to recognize that TGF-β1 activity vital to collagen synthesis is regulated by two distinct signaling mechanisms, the canonical Smad2,3 and non-canonical TAK1/MKK3/p38 pathways [19]. These pathways themselves may be influenced by ART. For example, the nuclear signaling adapter protein TRAF6 regulates both systems [20], and signaling through TAK1/MKK3/p38 includes proinflammatory cytokines that are elevated in HIV infection and induce TGF-β1 [21,22]. But TRAF6 function is modulated by immunoproteasome degradation. We found that low levels of RTV and certain other PIs specifically block immunoproteasome as opposed to constitutive proteasome subunit formation, thereby protecting TRAF6 from intracellular degradation and increasing the activity of cytokines dependent on TRAF6 function, which would include TGF-β1 [23]. The TAK1/MKK3/p38 pathway also encompasses a negative feedback loop involving microtubule-associated protein 1 light chain 3 (LC3)/Beclin-1. Inactivation of LC3/Beclin-1, critical to autophagy-dependent collagen degradation [24–27], led to a 3-fold increase in fibrosis in a murine cardiomyopathy model [25]. We hypothesized that antiretrovirals affecting that pathway could similarly interfere with collagen autophagy and promote fibrosis.

For the experiments described in this report we utilized two sets of transgenic mice, one with targeted deletion of TGF-β1 in megakaryocytes/platelets (PF4CreTgfb1^flox/flox^) [15], and another deficient in autophagosome formation, and thus unable to support collage autophagy (LC3^-/-^) [27], to document the importance of platelet TGF-β1 and collagen autophagy in RTV-associated cardiac dysfunction and fibrosis. We sought to model a novel intervention for ART-linked cardiac fibrosis, based on a mimetic of the anti-fibrotic effects of the type 1 inducible heme oxygenase (HO-1, also referred to as HO-I)/endogenous carbon monoxide (CO) pathway. It is known that low levels of exogenous CO can suppress basal and TGF-β1-stimulated collagen expression in mouse kidney via TAK1/MKK3/p38 signaling [26]. We here demonstrate that inhaled CO can mitigate RTV-induced cardiac fibrosis in conjunction with promotion of autophagy and macrophage polarization from proinflammatory to regulatory subsets, suggesting novel treatment approaches to HIV/ART-linked CVD.

## Materials and methods

### Mice and treatment

Three genotypes of mice were used: C57Bl/6 wt mice; mice with specific deletion of TGF-β1 in platelets (PF4CreTgfb1^flox/flox^) [15]; and autophagy-defective mice (LC3^-/-^) [27]. The PF4CreTgfb1^flox/flox^ mice were backcrossed for at least 10 generations on a C57Bl/6 background. For all genotypes, one group received daily intraperitoneal injections of vehicle (1% DMSO in PBS) or ritonavir (RTV; 10mg/kg body weight in vehicle) for 8 weeks. For all treatments, n = 6/group, unless otherwise noted.

### Carbon monoxide exposure

1% CO mixed with room air was directed into a 3.7 ft^3^ plexiglass exposure chamber at a flow rate of 12 L/min. A CO analyzer (Interscan, Chatsworth, CA) was used to continuously maintain the CO level at 250 ppm. Mice were exposed to CO for 4 h daily along with intraperitoneal administration of vehicle or RTV. Mice not undergoing CO exposures were maintained in ambient air.

### Fibrosis quantification

Mice were sacrificed and portions of each heart were fixed, processed, and embedded in paraffin blocks, then sectioned and stained with Masson’s trichrome and specific stains for collagen type-I α1 and α-smooth muscle actin (SMA), to identify extracellular matrix (ECM) deposition and fibrosis. High magnification images were taken of the slides using an Aperio Slide Scanner. Cardiac interstitial fibrosis was assessed after collagen staining associated with major coronary vessels was removed using the eraser tool in Microsoft Paint software. The resulting images were imported into ImageJ software for quantification of positive blue-stained regions compared to the entire tissue area, permitting calculation of percent fibrosis. The settings for positive staining in ImageJ were determined by first examining the original image with perivascular collagen and adjusting the saturation, hue and brightness such that blue-stained perivascular collagen was recognized as a positive area. Edited images, without major blood vessels, were then analyzed. These techniques enabled us to assess cardiac fibrosis in tissue sections from pediatric HIV-infected patients vs. controls, as well as in murine models of cardiac pressure overload [15,18] and renal fibrosis in mouse kidney [24,26].

### TGF-β1 determinations

Murine blood samples were collected using our previously established method, with 0.1% sodium citrate as an anti-coagulant and 1 uM PGE1 to prevent in vitro platelet activation [15]. This enables preparation of plasma with minimal in vitro platelet release of α-granule contents, which include TGF-β1, thereby precluding artefacts of ex vivo platelet activation. We monitored sample quality by immunoblot measurement of two α-granule proteins, platelet factor 4 and thrombospondin. TGF-β1 was quantitated by ELISA using a TGF-β1 DuoSet kit (R&D Systems) with modifications, as plates were coated with mAb2401 overnight and recombinant TGF-β1 was used as a standard, with 2-fold dilutions to increase assay sensitivity, after converting latent to active cytokine by acidification. Active TGF-β1 was measured directly without acidification.

### Cardiac tissue harvesting, macrophage subset determination, and TGF-β1 signaling analysis

To harvest hearts for preparation of tissue sections and extracts, animals were euthanized by carbon dioxide overdose and hearts were excised aseptically into warm saline. They were halved longitudinally along the aorta to apex, with one part prepared for extraction of total CD45 leukocytes and flow cytometry for macrophage subset analysis per established procedures [28], and the other half fixed in 4% paraformaldehyde for histology and immunohistochemistry. In preparation for flow cytometry, the harvested tissues were finely minced and digested in Liberase TM (0.25 mg/ml; Roche), deoxyribonuclease I (0.1mg/ml; Invitrogen), and DIspase (0.8 mg/ml; Roche) at 37°C for 30 min. Digestion was stopped by adding a solution of 2% FBS, 5mM EDTA, in PBS. After red blood cell lysis, the cells were incubated in Fc block with CD16/CD32 (eBiosciences). Antibodies for flow cytometry analysis included: CD45 (clone I.3/2.3); CD11b (clone M1/70); F4/80 (clone BM8); MHC class II I-A/E (clone M5/114.15.2); CD206 (clone C068C2); and B7-H4 (clone HMH4-5G1). Stained cells were analyzed on a BD FACS Canto flow cytometer. All antibodies were purchased from Biolegend, except for CD11b (BD Biosciences). Analysis was done using FlowJo software (Tree Star).

### Tissue staining

Paraffin-embedded heart sections were evaluated for ECM content by staining with Masson’s trichrome and specific immunostains for collagen type-I alpha1 (Sigma), and αSMA (Biolegend), as well as phospho-Smad2 (EMD Millipore), phospho-p38, and phospho-JNK (Cell Signaling) staining for these signaling pathways. Nuclei were stained with DAPI (Life Technologies). Quantitation of phospho-stained nuclei was performed using ImageJ.

### Echocardiographic assessment of cardiac function

Echocardiographic measurements were used to evaluate cardiac function over time with mice under isoflurane anesthesia and at a constant body temperature of 36-37°C. Ultrasounds were performed using B- and M-mode imaging (Vevo 2100, VisualSonics, Inc.) on mice just before treatment, and at 4 and 8 weeks thereafter. Images of the heart in parasternal long and short axis views were obtained in B- and M-Mode, respectively. They were analyzed by VevoLab software using the LV Trace tool. Anterior and posterior wall thickness and ventricular diameter measurements were made in diastole and systole, allowing the program to compute cardiac indices such as stroke volume, ejection fraction, fractional shortening, and cardiac output, as described by our lab [15].

### Hyaluronic acid

Hyaluronic acid in banked plasma samples from our HIV-negative and HIV-positive postmenopausal cohorts was measured by an ELISA (BioSource, Inc.) following the manufacturer’s instructions. Demographics of the two cohorts are given in Results.

### Statistics

Analysis of cardiac function and macrophage subsets involved 2-tailed Student’s t test. Correlations between measures of cardiac functions and TGF-β1 involved linear regression analyses. A Poisson regression model was used to determine association between categories of anti-HIV therapies and hyaluronic acid levels.

## Study approvals

Animal studies were approved by the Institutional Animal Care and Use Committee of OMRF and WCMC, and patient samples were obtained through study approval by the Institutional Review Board of WCMC.

## Ethical statement

All individuals were de-identified prior to being assayed. Written, informed consent was obtained from all study participants. This study was approved by the Institutional Review Boards of the Cornell and the ethical committees where subjects were recruited.

## Results

### Importance of platelet-derived TGF-β1 in protease inhibitor-based cardiac dysfunction

**Fig 1:**
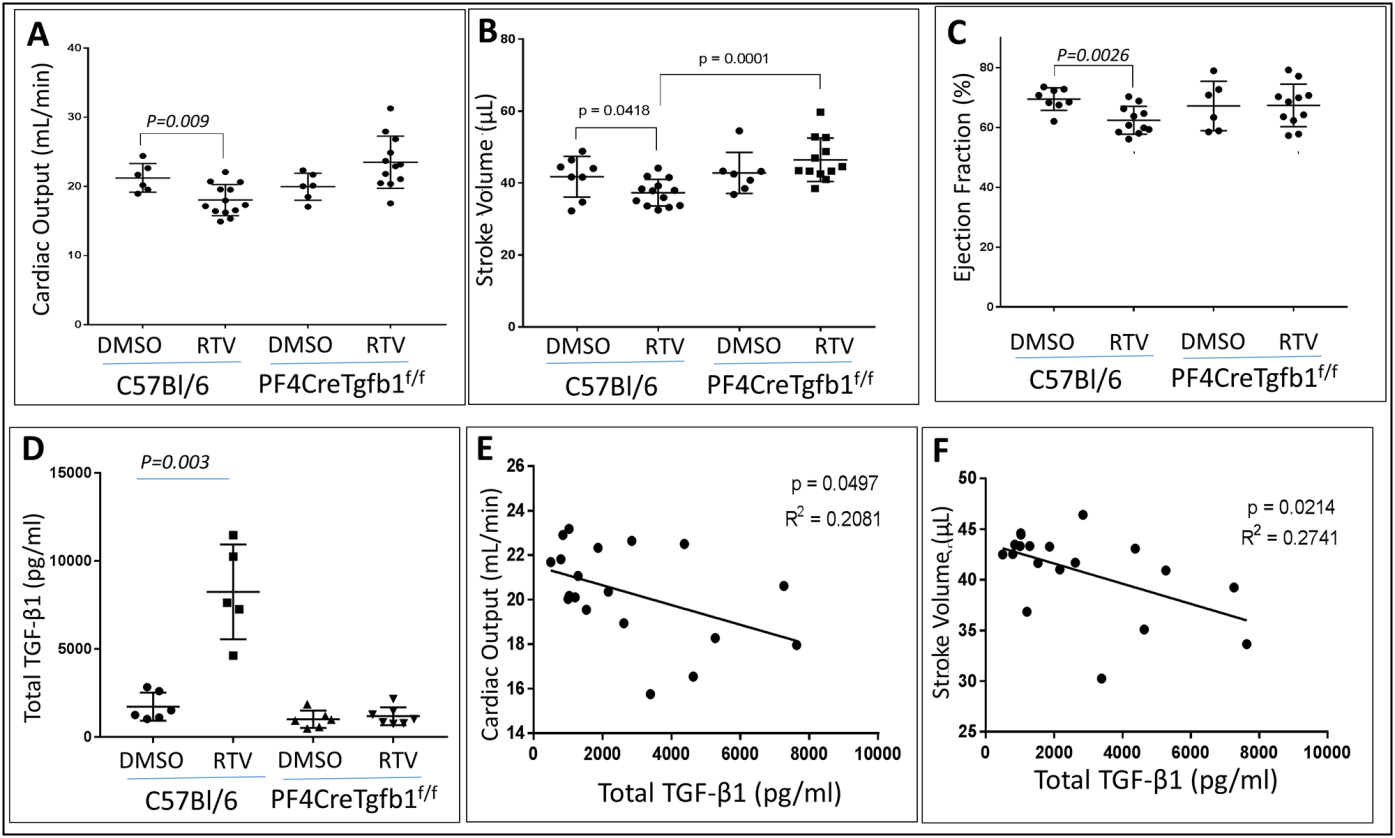
Effect of RTV on cardiac function and plasma TGF-β1. Cardiac output **(A**), stroke volume (**B**), and ejection fraction **(C)** were measured by echocardiography. Plasma TGF-β1 levels were measured by ELISA. Cardiac output, stroke volume, and ejection fraction were depressed by exposure to RTV, 10mg/kg daily over 8 weeks, vs. mice administered vehicle (DMSO). Plasma TGF-β1 levels were measured by ELISA (**D**). RTV exposure elevated TGF-β1 levels in wt mice but not in platelet TGF-β1 deficient PF4Cre Tgfb1^flox/flox^ mice (**D**). Cardiac output (**E**) and stroke volume (**F**) were negatively correlated with TGF-β1 levels.

To assess whether pharmacological doses of RTV initiate cardiac dysfunction dependent upon platelet TGF-β1, we treated C57BL/6 wt mice and mice with targeted deletion of TGF-β1 in megakaryocytes/platelets (PF4CreTgfb1^flox/flox^) with vehicle (1% DMSO in PBS) or RTV (10 mg/kg in vehicle) intraperitoneally daily for 8 weeks. A decrease in cardiac function indices, including cardiac output, ejection fraction, and stroke volume, was prominent in wt mice exposed to RTV, while PF4CreTgfb1^flox/flox^ mice were protected from deterioration of these functions (Fig 1A–1C). Total TGF-β1 levels were increased nearly 5-fold in plasma of RTV-treated wt mice vs. vehicle controls; in contrast, there was no increase in TGF-β1 levels in PF4CreTgfb1^flox/flox^ mice treated with RTV vs. vehicle (Fig 1D). Linear regression analysis of cardiac output and stroke volume showed a negative correlation with TGF-β1 levels for RTV-exposed wt mice (Fig 1E and 1F).

### Platelet-derived TGF-β1 contributes to protease inhibitor-induced cardiac fibrosis

**Fig 2:**
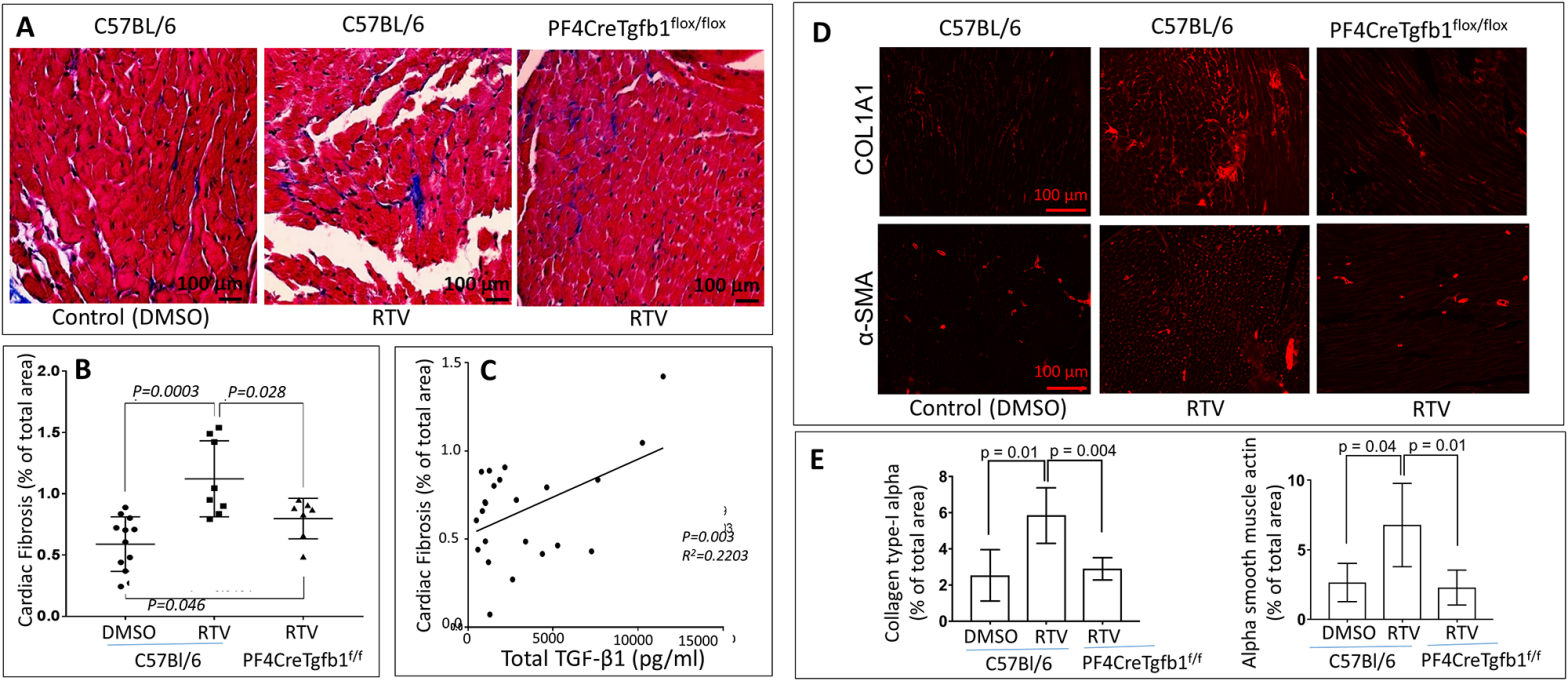
PF4CreTgfb1^flox/flox^ mice are partially protected from RTV-mediated cardiac dysfunction. Wt and platelet-deficient TGF-β1 PF4CreTGFb1^flox/flox^ mice were treated with RTV or vehicle (DMSO) for 8 weeks. Hearts were harvested and sections stained with Masson trichrome to evaluate for fibrosis (**A**), with staining quantified by ImageJ analysis (**B**). RTV exposure led to a marked increase in cardiac fibrosis in wt but not platelet-TGFβ1 deficient mice. Cardiac fibrosis correlated with plasma levels of TGF-β1 (**C**). These effects were paralleled by changes in collagen type-1 α1 and αSMA, as shown in representative cardiac sections (**D**) and by ImageJ analysis (**E**).

RTV-exposed wt and PF4CreTgfb1^flox/flox^ mice were sacrificed after eight weeks. Fibrotic areas in the heart were identified by staining with Masson’s trichrome (Fig 2A) as well as staining for collagen type-1 α1 and αSMA (Fig 2D), and quantified using an ImageJ program. Higher levels of fibrosis were seen in hearts of RTV-exposed wt vs. PF4CreTgfb1^flox/flox^ mice (Fig 2B). The degree of cardiac fibrosis correlated directly with total plasma TGF-β1 levels (Fig 2C). This difference was paralleled by higher levels of expression of collagen type-I α1 (COL1A1) and αSMA in wt mice treated with RTV compared to RTV-treated PF4CreTgfb1flox/flox mice (Fig 2D and 2E).

### Inhaled CO protects against ritonavir-mediated cardiac fibrosis

**Fig 3:**
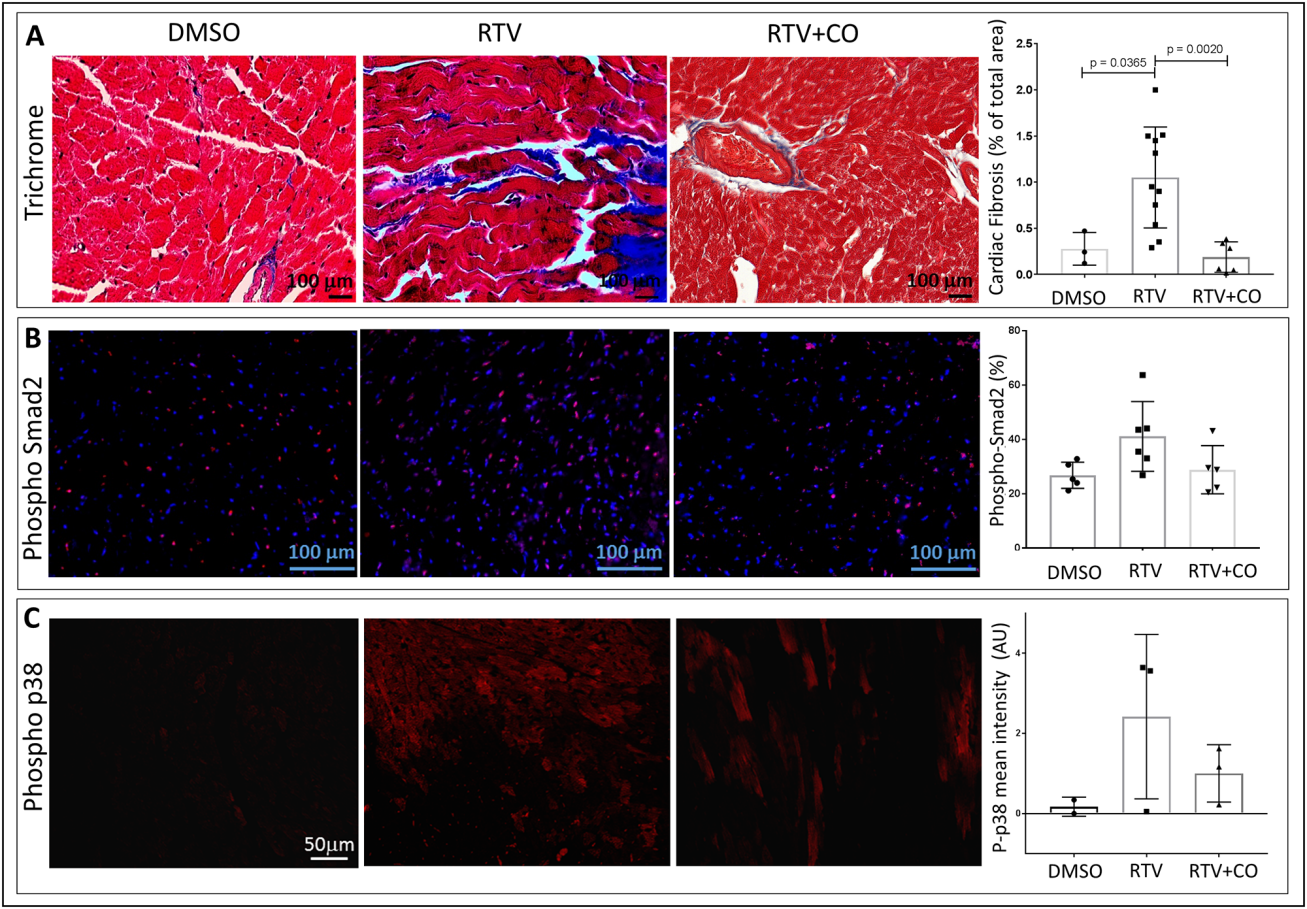
CO suppressed RTV-induced cardiac fibrosis and TGF-β1 signaling. Wt mice were exposed to RTV and inhaled CO (250ppm) or ambient air for 4 hrs daily for 8 weeks. CO markedly reduced RTV-induced fibrosis, assessed as described in (**A**). CO also reduced phospho-Smad2 **(B)** and phospho-p38 (p-p38) **(C)** staining, which was elevated over controls by RTV exposure, indicating effects on both canonical and non-canonical TGF-β1 signaling pathways, respectively.

CO is generated endogenously in mammalian cells through the catalysis of heme by HO, and the stress-inducible form HO-I has a key physiologic role in protecting against oxidative stress [26]. Low dose exogenous CO can fully substitute for the cytoprotective effects of HO-I in a variety of in vitro and in vivo models. For example, we had shown that inhaled CO blocked renal fibrosis in a murine model of unilateral ureteral obstruction (UUO), together with decreased expression of TGF-β receptor types I and II [26,27]. This occurred at doses of 250-1500ppm which, at least in the short-term, have been used without adverse effects in human and animal studies [26,29]. In addition, exogenous CO has anti-apoptotic [30] and anti-proliferative [31] properties, and can promote collagen autophagy [24]. With this background, two groups of wt mice were exposed to CO (250ppm) or ambient air in inhalation chambers for 4hrs after each RTV or vehicle injection over 8 weeks. CO dramatically blocked RTV-induced cardiac fibrosis (Fig 3A). This occurred in parallel with suppression of staining for phospho-Smad2 and phospho-p38 (Fig 3B and 3C), and phospho-JNK (data not shown), supporting a role for CO acting via canonical and non-canonical TGF-β1 signaling pathways. These changes are consistent with the anti-fibrotic effects of CO in our group’s UUO fibrosis model, as CO had no impact on fibrosis induction in Mkk3^-/-^ mice [26]. MKK3 is one of the immediate upstream MAPK kinases required for activation of p38 and JNK. CO also required p38 activation to protect against TNF-α-induced endothelial cell injury and oxidant-induced lung injury (reviewed in [26]).

### Protection against RTV-mediated cardiac fibrosis by CO is dependent on autophagy

**Fig 4:**
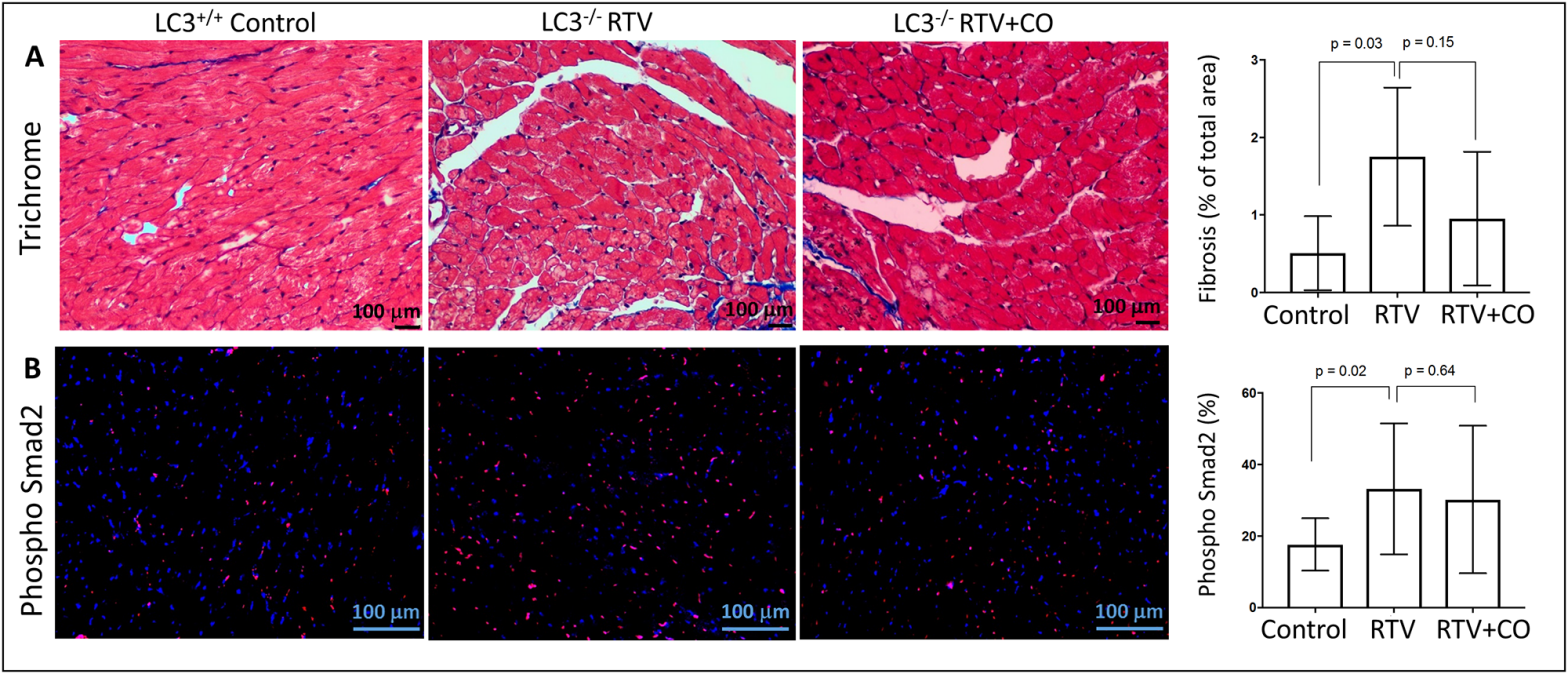
Autophagy deficient mice are resistant to the ability of CO to suppress RTV-associated cardiac fibrosis and TGF-β1 signaling. LC3^-/-^ mice were treated with RTV or RTV+CO for 8 weeks. **(A)** Cardiac fibrosis was evaluated by Masson’s trichrome staining; representative images of heart sections are shown. Quantification of interstitial fibrosis showed that CO had no effect on RTV-induced fibrosis in these mice (n = 5). **(B)** Phosph-Smad2 staining (red) of DAPI+ nuclei (blue). Merged images show phospho-Smad2 and DAPI double-positive nuclei (purple). Phospho-Smad2 positive nuclei were counted using the ImageJ program, which showed no decrease in phospho-Smad2 in the CO/RTV group compared to mice exposed to RTV alone (n = 5).

In the UUO model the degree of kidney fibrosis inversely correlates with collagen autophagy, assessed by induction of Beclin-1 and LC3 [24,26,27]. LC3 is a structural component of autophagosomes, and is a widely used autophagy marker [24]. We found that suppression of RTV-mediated cardiac fibrosis by CO was similarly dependent on autophagy. Wt and LC3^-/-^ mice showed RTV-induced increases in cardiac collagen deposition, together with increased pSmad2 staining, but these changes were not mitigated by CO inhalation in the LC3^-/-^ mice (Fig 4).

### Role of macrophage polarization in ritonavir-associated cardiac fibrosis, and its suppression by CO

**Fig 5:**
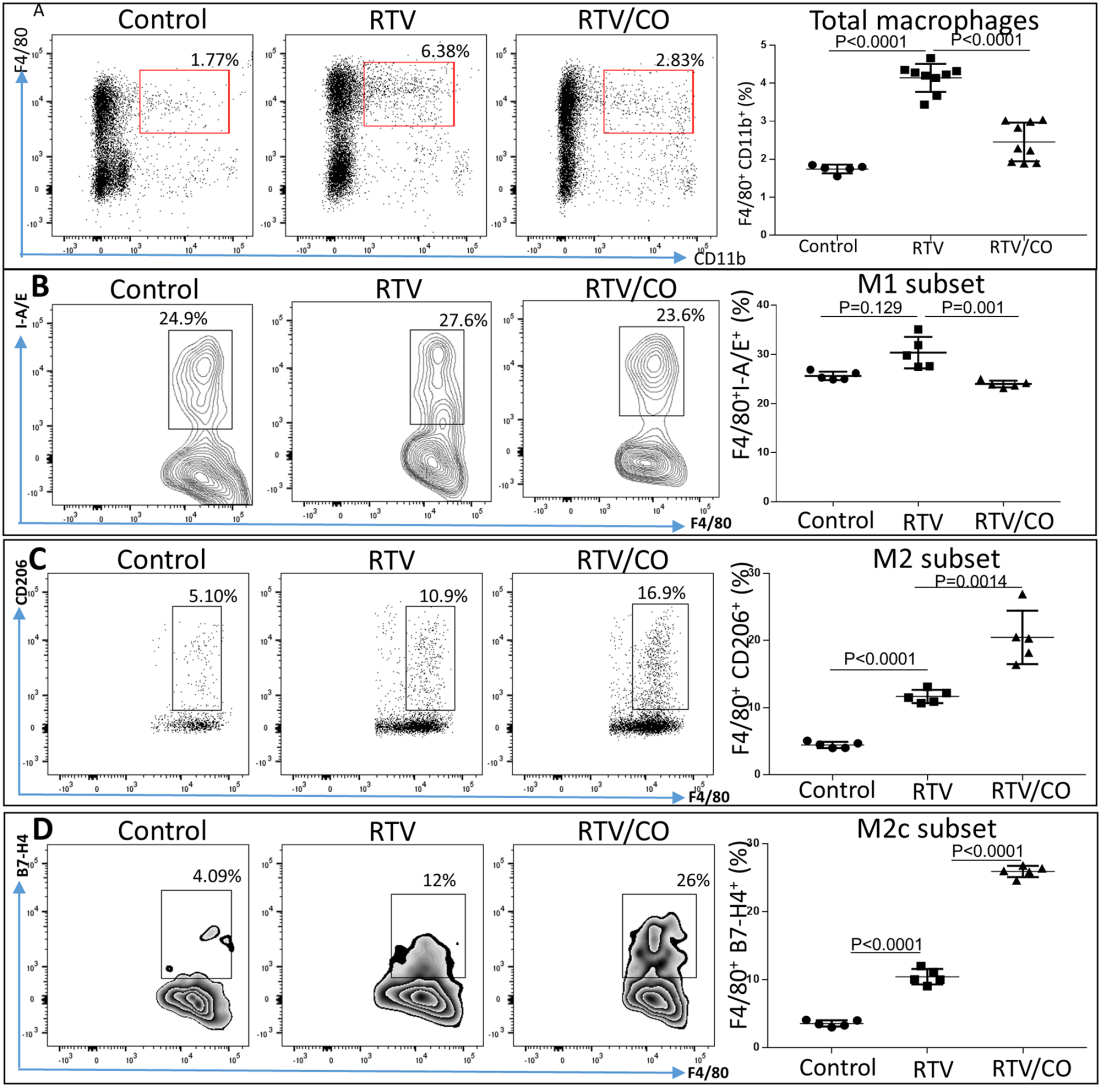
CO modulates macrophage polarization in RTV-treated heart muscle. **(A)** Total F4/80^+^ CD11b^+^ macrophages, expressed as a percentage of CD45+ leukocytes, were elevated by RTV exposure and reduced to near basal levels by CO. **(B)** RTV had little effect on the pro-inflammatory F4/80^+^ I-A/E^+^ M1 subset, which was reduced to basal levels by CO. (**C**) RTV increased the number of F4/80^+^ CD206+ M2 cells as a proportion of total macrophages, an effect markedly augmented by CO. The CO-mediated alteration in M2 cells predominantly involved augmentation of regulatory, anti-inflammatory F4/80^+^ B7-H4^+^ M2c cells **(D)**.

Pan-neutralization of TGF-β fails to inhibit or reverse fibrosis in many murine models and clinical trials. It has been proposed that this lack of response relates to divergent TGF-β1-dependent pathways which can either augment collagen synthesis or promote its degradation [32]. Macrophage polarization may be involved in these processes through loss of the anti-fibrotic effect of TGF-β1 produced by the M2c regulatory macrophage subset acting via TAK1/MKK3/p38 signaling and promotion of autophagy [32,33]. In advanced HIV disease, and in the presence of ART, both pro-inflammatory M1 cells and regulatory M2c cells proliferate [34]. We hypothesized that the M2c subset is linked to the anti-fibrotic activity of CO, and assessed macrophage phenotypes in the hearts of mice exposed to RTV vs. RTV plus CO. RTV led to a 3.6-fold increase in total macrophages, quantitated as a fraction of total CD45+ mononuclear cells in homogenized hearts, which was reduced to near basal levels by CO (Fig 5A). RTV had no effect on levels of M1 cells (Fig 5B) but increased M2 macrophages (Fig 5C). Co-exposure of RTV treated me to CO led to a significant decrease in pro-inflammatory M1 cells (Fig 5B, p = 0.001), and dramatically increased the M2 subset, consisting predominantly of the regulatory, anti-inflammatory M2c subset (Fig 5D). This is the first time that CO has been documented to influence macrophage polarization.

### Correlation of circulating biomarkers for LV dysfunction and fibrosis with ritonavir-based ART in HIV-infected individuals

Neither HIV nor HIV proteins were utilized in our model, as there is no rodent system to reproduce the undetectable levels of replication-competent HIV or circulating soluble HIV antigens characteristic of HIV/ART. Given this limitation, we examined changes in biomarkers for LV dysfunction and autophagy on HIV+ individuals on various ART regimens. We utilized banked plasmas from an observational cohort of 100 HIV+ and 100 HIV- postmenopausal women followed longitudinally for two years [35]. Subjects were comparable in age (mean 55 years), ethnicity, tobacco, ethanol, hormone and illicit drug use, hypertension, and endocrine disease. Among the HIV+, there were approximately equal numbers in three groups: HIV treatment-naïve or off treatment; RTV-containing ART; and non-nucleoside reverse transcriptase inhibitor (NNRTI)-based ART. There were no differences in total time on ART or estimated total time of HIV infection [35]. We reported that the N-terminal fragment of the prohormone B-type natriuretic peptide (NT-proBNP), a marker for LV dysfunction and a prognostic factor for CVD in the general population [36] as well as in HIV+ individuals [37], correlated with RTV treatment [18]. Only the HIV+ women on RTV-based ART showed an increase in the highest quartile of NT-proBNP [18]. We now find that those data parallel an RTV-associated increase in a biomarker for fibrosis, plasma hyaluronic acid [38]. HIV infection was linked to elevated hyaluronic acid in the treatment-naïve group, and this was not suppressed by RTV (Table 1). In contrast, patients receiving a non-PI-based regimen had no significant elevation in this marker compared to HIV^-^ negative women (Table 1).

**Table 1:** Plasma hyaluronic acid levels in HIV+ postmenopausal women receiving no, or various, anti-retroviral regimens, vs. HIV- postmenopausal controls.

Status	N	Hyaluronic acid (mean ±SD, ng/ml)	p-value
**HIV-**	**27**	**32.6 ±20.0**	**-**
**HIV+, no ART**	**27**	**56.7 ±42.4**	**0.007**
**HIV+, non-PI-based ART**	**30**	**38.4 ±29.6**	**0.17**
**HIV+, ritonavir-based ART**	**20**	**65.0 ±63.3**	**0.009**

Hyaluronic acid was measured in plasmas from postmenopausal women, either HIV seronegative, HIV-positive on no antiretroviral (ART) therapy, or HIV-positive on a stable ART regimen for ≥two years. The later involved either a ritonavir-boosted protease inhibitor (PI) or a non-PI based regimen (primarily non-nucleoside reverse transcriptase inhibitor- based).

## Discussion

Our studies document the importance of TGF-β1, derived from platelets activated by the HIV protease inhibitor ritonavir, in cardiac dysfunction that is potentially mediated by fibrosis. Fibrosis in our murine model was accompanied by polarization of cardiac macrophages toward a pro-inflammatory subset, consistent with the strong association between sCD163, a plasma marker of macrophage activation, and arterial inflammation and fibrosis in HIV+ individuals on ART [39,40]. These changes were paralleled by a marked rise in circulating TGF-β1. The failure of an earlier study to document more than a trend to increased TGF-β1 in RTV-exposed mice could relate to methods of plasma preparation which do not block non-specific ex vivo activation of TGF-β [13]. This may also be an important confounding factor in clinical studies of fibrotic biomarkers in HIV-associated CVD, as we have recently demonstrated the importance of measures to suppress ex vivo platelet activation, and directly quantify active TGF-β1 levels, in evaluation of circulating TGF-β1 in humans, methods not commonly employed [41].

The RTV-associated changes we report may serve as a model to evaluate other antiretrovirals, both PIs and drugs of other anti-HIV classes which, like RTV, also activate platelets and have been linked to accelerated CVD in humans [17,42]. The minimal fibrosis and cardiac dysfunction in RTV-exposed PF4CreTgfb1^flox/flox^ mice that are deficient in platelet TGF-β1 indicates that RTV-associated elevation of TGF-β1 levels and related signaling was not simply a reaction to tissue damage from a cardiotoxic drug. In terms of possible confounding factors, CVD linked to RTV and other first generation PIs is accompanied by changes in lipid metabolism [42,43], but dyslipidemia was not a factor in at least two prior rodent models of RTV-driven cardiac disease [11,13], nor was it involved in the increased CVD risk related to more contemporary PIs such as RTV-boosted darunavir/RTV in humans [42].

In our pathophysiologic model for ART-linked CVD, one apparent clinical exception bears scrutiny: RTV-boosted atazanavir, a contemporary PI regimen linked to hyperlipidemia [42], has not been implicated in CVD [42]. However, unlike RTV, atazanavir decreases platelet reactivity, and increases autophagy [44,45]. Examination of such opposing processes by a single drug may be a critical consideration in defining, and perhaps predicting, the influence of a specific antiretroviral drug or ART regimen on development of CVD. For example, duration of RTV-boosted PI treatment is a significant association for CVD among HIV-infected adolescents [3], but when considering PIs as a class they appear to be “cardioprotective” for HIV-infected children [46].

In terms of modeling therapeutics for ART-linked CVD, it is important to note that both pro-fibrotic and extracellular matrix-preserving phenomena related to TGF-β1 have been well described in cardiac injury [47]. One group emphasized that the cellular source of TGF-β1 “dictates its activity [so that] it remains unclear whether antagonism of the TGF-β1 signaling pathway will prove beneficial in humans” [48]. Indeed, the results of our study suggesting involvement of non-canonical TGF-β1 signaling pathways, which have been associated with macrophage polarization toward an anti-inflammatory subset and induction of autophagy, indicate that pan-TGF-β neutralization could be counter-productive clinically. Recent clinical trials of several such agents in the general population have been disappointing [32].

We utilized exogenous CO as a proof-of-concept to support future work targeting those pathways by which HO-I/endogenous CO appears to act. This is important as, apart from pragmatic considerations, chronic inhalation of CO, even at very low doses, may have unacceptable toxicities. Although a multi-center clinical study (NCT01214187) for the treatment of idiopathic pulmonary fibrosis with CO inhalation, 250ppm two times weekly, two hours per dose, for 12 weeks, has been conducted, and brief inhalation of 1500ppm CO 20 times per day for a week produced no cardiovascular effects [29], chronic exposure to even very low dose inhaled CO can lead to significant myocardial damage in rodents and humans [49, 50]. But a key regulator of the expression of genes coding for the majority of endogenous anti-oxidant and anti-inflammatory proteins linked to amelioration of tissue fibrosis is nuclear factor erythroid 2-related factor (Nrf2), and many of its functions may be mimicked by CO, apart from HO-I [51]. This is an important consideration as RTV, at least at supra-pharmacologic concentrations, induces HO-1 in macrophages [52]. In addition, while HO-I is generally considered to be an anti-inflammatory mediator, recent studies demonstrate that it can also define pre-stimulation thresholds for pro-inflammatory processes such as NFκB amplification in macrophages, leading to chronic metabolic “cold” inflammation [53]. Earlier work suggesting HO-I as the effector in blocking certain inflammation-mediated metabolic abnormalities may instead have involved alternate pathways which upregulated Nrf2 [53].

Linkage of RTV and other HIV PIs to increases in oxidative stress, together with dysregulation of the ubiquitin-proteasome system [54], which was first reported by our group in association with the ability of RTV to block degradation of TRAF-6, critical to the function of TGF-β1 and other proinflammatory cytokines [23], strengthens the argument for use of Nrf2 modifying agents in modifying HIV/ART-associated tissue fibrosis. For example, S-adenosylmethionine can treat hepatic fibrosis in mice through induction of Nrf2-mediated pathways [55, 56]. Other potential modifiers of TGF-β1 signaling such as pirfenidone [57], Smad7 overexpression [32], and activation of AKT-mTOR [32], along with inhibitors of connective tissue growth factor (CTGF, CCN2), a central mediator of fibrosis [58], should also be considered.

## Supporting information

S1 Table NC3Rs ARRIVE guidelines checklist. (PDF)
